# METTL3‐mediated m^6^A modification of circPRKAR1B promotes Crohn's colitis by inducing pyroptosis via autophagy inhibition

**DOI:** 10.1002/ctm2.1405

**Published:** 2023-09-07

**Authors:** Jie Zhao, Zhibin Zhao, Pu Ying, Yan Zhou, Ziwei Xu, Honggang Wang, Liming Tang

**Affiliations:** ^1^ Department of Gastrointestinal Surgery Affiliated Changzhou No.2 People's Hospital of Nanjing Medical University, Changzhou Medical Center, Nanjing Medical University Changzhou P. R. China; ^2^ Department of Gastroenterology Affiliated Taizhou People's Hospital of Nanjing Medical University, Taizhou School of Clinical Medicine, Nanjing Medical University Taizhou P. R. China; ^3^ Department of Orthopedics Changshu Hospital Affiliated to Nanjing University of Chinese Medicine Changshu P. R. China; ^4^ Department of General Surgery First Affiliated Hospital of Nanjing Medical University Nanjing P. R. China; ^5^ Department of General Surgery Affiliated Taizhou People's Hospital of Nanjing Medical University, Taizhou School of Clinical Medicine, Nanjing Medical University Taizhou P. R. China

**Keywords:** autophagy, circPRKAR1B, Crohn's disease, m^6^A modification, pyroptosis, SPTBN1

## Abstract

**Background:**

The roles of circRNA and N6‐methyladenosine (m^6^A) methylation in Crohn's disease (CD) have drawn much attention. Therefore, this investigation aimed to discover how the m^6^A modification of circRNAs contributes to CD progression.

**Methods:**

The study performed circRNA sequencing on colon samples from four CD patients and four normal controls (NCs) to screen for dysregulated circRNAs. Quantitative real‐time polymerase chain reaction (qRT‐PCR) was performed to validate the candidate circRNA expression and determine its correlation to CD‐associated inflammatory indicators. In vivo and in vitro investigations were conducted to examine the functions and pathways of circPRKAR1B in CD, besides investigating the m^6^A modification role in circRNA expression modulation.

**Results:**

The RNA‐seq revealed that hsa_circ_0008039 (circPRKAR1B) was the most significant upregulated circRNA and was identified as the candidate circRNA for further examinations. Relative circPRKAR1B expression was significantly upregulated in CD colon tissues and closely related to CD‐associated inflammatory indices. The circPRKAR1B expression and function were regulated by methyltransferase‐like 3 (METTL3)‐mediated m^6^A methylation. In vitro studies indicated that circPRKAR1B promoted pyroptosis mediated by NLRP3 inflammasome (NLRP3; nucleotide‐binding oligomerization domain, leucine‐rich repeat and pyrin domain‐containing 3) and impaired autophagy by interacting with the RNA‐binding protein (RBP) SPTBN1, (SPTBN1; spectrin beta, non‐erythrocytic 1). The in vivo investigations revealed the treatment effects of si‐circPRKAR1B and si‐METTL3 in colitis models of IL‐10‐deficient mice.

**Conclusion:**

Our study reveals that METTL3‐mediated m^6^A modification of circPRKAR1B promotes Crohn's colitis by aggravating NLRP3 inflammasome‐mediated pyroptosis via autophagy impairment in colonic epithelial cells.

## Graphical abstract

- METTL3‐mediated m6A modification of circPRKAR1B involves in the progression of Crohn's colitis.
- CircPRKAR1B promotes Crohn's colitis by modulation of autophagy and NLRP3 inflammasome‐mediated pyroptosis.
- The known down of circPRKAR1B or METTL3 alleviates experimental colitis of IL‐10 knock‐out mice.

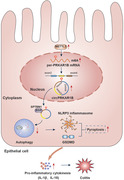

## 1 INTRODUCTION

Crohn's disease (CD) is a chronic gastrointestinal tract inflammatory disease classified as an inflammatory bowel disease (IBD) subtype. It can affect all gastrointestinal tract parts, particularly the terminal ileum and colon. The CD is characterized by immune‐mediated inflammation, with a rising incidence globally.^1^ Although the precise CD pathogenesis is unclear, it may involve a complex interplay among environmental factors, genetic predisposition, gut microbiota alteration and dysregulated immunology.^2^, ^3^

Circular RNA (circRNA) is an emerging endogenous RNA species characterized by a closed‐loop structure that is covalently bonded and lacks a 5′−3′ polarity.^4^ The circRNAs modulate diverse physiological and pathological mechanisms,^5^ and their differential expression during disease progression indicates their significance.^6^, ^7^ Moreover, circRNAs function through many putative mechanisms; they can serve as miRNA “sponges,” react with RNA‐binding proteins (RBPs), engage in post‐transcriptional gene regulation and encode proteins.^8^ N6‐methyladenosine (m^6^A) is a chemical modification in various RNA species and can regulate several biological mechanisms including RNA degradation, circRNA loop formation, transcript splicing and protein translation.^9^ Furthermore, m^6^A modification is abundant in many circRNAs, which can drive circRNA translation like the internal ribosome entry site.^10^ Therefore, the role of circRNAs and m^6^A methylation modification in CD has drawn much attention.

Based on circRNA‐sequencing (circRNA‐seq), this study determined the most significant upregulated circRNA, hsa_circ_0008039 (circPRKAR1B), as a circRNA candidate. Subsequent quantitative real‐time polymerase chain reaction (qRT‐PCR) investigations verified that the circPRKAR1B upregulation in CD‐affected colon tissues was strongly correlated to CD‐associated inflammatory markers. These outcomes revealed that circPRKAR1B is a circRNA closely linked to CD. Mechanistically, circPRKAR1B functions within the colonic epithelial cells by interacting with the RNA‐binding protein (RBP) SPTBN1 (SPTBN1; spectrin beta, non‐erythrocytic 1). This interaction modulates autophagy and pyroptosis mediated by NLRP3 inflammasome (NLRP3; nucleotide‐binding oligomerization domain, leucine‐rich repeat and pyrin domain‐containing 3). Additionally, In vivo experiments verified that knocking down circPRKAR1B and methyltransferase‐like 3 (METTL3) ameliorated experimental colitis in mice with IL‐10‐knockout (IL‐10‐KO). Herein, we identified the functions and underlying mechanisms of circPRKAR1B and m^6^A modification in Crohn's colitis.

## 2 MATERIALS AND METHODS

### 2.1 Patient samples

Colon specimens were acquired from 77 individuals with colonic or ileocolonic CD (4 in the discovery and 73 in the validation sets) who underwent elective colectomy at the First Affiliated Hospital of Nanjing Medical University between 2021 and 2023. The investigation followed the Helsinki Declaration and was authorized by the First Affiliated Hospital of Nanjing Medical University's Ethics Committee. After surgical resection, soybean‐sized samples comprising lamina propria (LP) were obtained, rapidly frozen in liquid nitrogen, and preserved at −80°C for subsequent examination. Additionally, normal colonic samples were acquired from 77 age‐matched patients with matched sex who underwent radical colectomy for colonic carcinoma. The inclusion criteria for CD patients were individuals aged between 18 and 65 years, diagnosed with a colonic or ileocolonic CD diagnosis and having signed informed consent for elective colectomy. The exclusion criteria were individuals having malignancies, ileal or ileocolonic CD with diffuse small bowel injuries, and additional autoimmune disorders, receiving emergency operations (due to complications including intestinal perforation or haemorrhage), and unwillingness to participate. The study evaluated CD‐associated inflammatory markers before surgery, including C‐reactive protein (CRP), Crohn's disease activity index (CDAI)^11^ and SES‐CD.^12^ Table 1 contains comprehensive demographic information of the participants, including age and gender.

**TABLE 1:** Patient demographics for the cohort.

	Discovery set (RNA‐seq)			Validation set (qRT‐PCR)		
	CD	NC	*p*‐value	CD	NC	*p*‐value
Number	4	4	–	73	73	–
Age (years)	35.8 ± 2.9	36.5 ± 3.4	0.765	40.2 ± 6.4	40.3 ± 5.7	0.982
Gender, n (%)	–	–	1.000	–	–	0.228
Male	3	3	–	50	43	–
Female	1	1	–	23	30	–
BMI (kg/m^2^)	21.1 ± 1.2	21.2 ± 0.6	0.886	20.4 ± 2.4	21.0 ± 2.2	0.725
Disease location, n (%)	–	–	–	–	–	–
Colonic	1	–	–	28	–	–
Ileocolic	3	–	–	45	–	–
Disease duration (months)	46.5 ± 5.6	–	–	53.0 ± 27.3	–	–

Abbreviations: BMI, body mass index; CD, Crohn's disease; NC, normal control.

### 2.2 Whole‐transcriptome sequencing (RNA‐seq)

Four inflamed colon tissues from CD patients and four matched ones from colonic carcinoma patients were used for RNA‐seq. The study employed the Isolation Kit of mirVana miRNA (Ambion) to obtain total RNA, following the protocols. RNA integrity was assessed using the Agilent 2100 Bioanalyzer (Agilent Technologies). The subsequent analysis was conducted on specimens with an RNA integrity number ≥7. The libraries were prepared using TruSeq Stranded Total RNA with Ribo‐Zero Gold, per the protocols. Subsequently, the libraries were sequenced employing the Illumina platform (HiSeq™ 2500) to produce paired‐end reads of 150 and 125 bp. Oebiotech (Shanghai OEbiotech Co., Ltd.) performed RNA‐seq and data analysis.

### 2.3 Animal experiments

Experimental colitis models were established employing IL‐10‐KO mice (C57BL/6; GemPharmatech Co., Ltd.) raised under specific pathogen‐free circumstances. The Laboratory Animal Center at Nantong University approved all animal experimental procedures (No. S20200323‐289). Twelve‐week‐old wild‐type (WT) and IL‐10‐KO mice were allocated randomly to four groups (n = 5 per group): (1) the WT group; (2) the IL‐10 KO + si‐control group (administrated saline as a placebo via enema once); (3) the IL‐10 KO + si‐circPRKAR1B group (administrated 200 μL of 1e11 vg adeno‐associated virus (AAV) vector carrying si‐circPRKAR1B via enema once); and (4) the IL‐10 KO + si‐METTL3 group (administrated 200 μL of 1e11 vg AAV vector carrying si‐METTL3 via enema once). The study evaluated the disease activity index (DAI), including rectal prolapse, occult faecal blood and ruffled fur, following the previously described conditions.^13^ Following one‐week drug administration, the mice were humanely euthanized, and the complete colon was obtained to measure its length, besides obtaining the proximal colon tissues for subsequent examinations.

### 2.4 Histological analysis

The proximal colon segments were subjected to haematoxylin and eosin (HE) staining to assess histological inflammation. To ensure impartial evaluation, two independent pathologists were invited to score the inflammatory levels using the assessment standards as defined by Singh et al.^14^ Concisely, a score of 0−4 was assigned depending on the following measures: 0 indicated no deviation from healthy tissue; 1 denoted minimal hyperplasia with one or few multifocal mononuclear cell infiltrates in the LP and no mucus depletion; 2 presented lesions exhibiting multifocal mild inflammatory cell infiltrates in the LP with no sub‐mucosal inflammation; 3 showed lesions displaying moderate inflammation and epithelial hyperplasia and 4 revealed lesions involving extensive intestinal sections affected by inflammation.

### 2.5 Proinflammatory cytokine measurement

An enzyme‐linked immunosorbent assay (ELISA) kit (R&D Systems) was utilized to detect proinflammatory cytokine levels in the proximal colons or epithelial supernatants, precisely tumour necrosis factor‐α (TNF‐α), interferon‐γ (IFN‐γ), interleukin (IL)‐1β/18/17. Briefly, colon tissues were washed, homogenized and stored overnight at ≤−20°C. Following two freeze‐thaw rounds and centrifugation, the collected supernatant was subjected to ELISA per the protocols. NCM460 cells grouped post‐intervention were seeded on a 24‐well plate and centrifuged, detecting cytokine levels in the supernatant with ELISA.

### 2.6 Analysis of epithelial apoptosis

The study assessed epithelial apoptosis (21) employing the terminal deoxynucleotidyl transferase dUTP nick end labeling (TUNEL) assay through In Situ Cell Death Detection Kits (Roche).Briefly, the colon sections underwent permeabilization, washing, staining and counterstaining with 40,6‐diamidino‐2‐phenylindole (DAPI) (Servicebio). Subsequently, the sections were washed and mounted in 50% glycerol. The TUNEL‐positive cell or field was quantified through manual calculation, capturing images utilizing a confocal microscope (Olympus).

### 2.7 Immunofluorescence staining

The colon tissue immunofluorescence staining procedures^15^ were performed as follows: the sections were fixed, washed and blocked using a 5% normal goat serum solution. Subsequently, primary antibodies were introduced, and the sections were incubated overnight at 4°C. Subsequently, the segments were washed and incubated with corresponding secondary antibodies (Cell Signalling Technology) for 1 h at room temperature. The specimens were counterstained with DAPI to facilitate nuclear visualization, acquiring images with a confocal microscope (Olympus). The study conducted an immunofluorescence analysis of NCM460 or HcoEpic cells.^16^ Briefly, NCM460 cells were fixed, permeabilized and blocked. Subsequently, the cells were incubated with the primary antibodies, anti‐LC3B (Abcam: ab192890), anti‐GSDMD (Abcam: ab219800), anti‐NLRP3 (Abcam: ab4207) and anti‐Caspase 1 (Sigma: AB1871), at 4°C overnight. Then, the cells were washed, incubated with the appropriate secondary antibodies and stained with DAPI to mark the nuclei. The samples were visualized using a confocal microscope (Carl Zeiss; Oberkochen).

### 2.8 Isolation of primary epithelial cells

Primary epithelial cell isolation from human colon samples was conducted^17^ by sectioning colon specimens into approximately .5 cm fragments, followed by incubation in a phosphate buffered saline solution containing 2 mmol/L of dithiothreitol and 1 mmol/L of EDTA, with a little shaking. The isolated epithelial cells were purified through density gradient centrifugation, employing a 20% and 40% Percoll‐RPMI solution to be used for further experiments.

### 2.9 Transmission (TEM) and scanning electron microscopy (SEM)

TEM was utilized to examine the autophagosome or autophagolysosome.^18^ Briefly, tissue or cell samples were fixed, post‐fixed, dehydrated, infiltrated in Epon812 and immersed in resin. The obtained ultrathin slices were then subjected to uranyl acetate and lead citrate staining and visualized and photographed utilizing a HITACHI HT7700 TEM. SEM was used to observe pyroptosis; samples were washed, fixed, dehydrated and treated with isoamyl acetate:ethanol (1:1) and isoamyl acetate for 10 min each and dried with a critical‐point drier. Afterward, the specimens were introduced into an ion sputter coater, where they underwent gold sputtering and were subsequently examined utilizing a HITACHI Regulus 8100 SEM.

### 2.10 Western blot analysis

The protein expression analysis was conducted through western blot analysis.^19^ Herein, we used radioimmunoprecipitation assay lysis buffer (Beyotime) to obtain proteins from colon tissues or cells. The protein level was assessed utilizing a bicinchoninic acid Protein Assay Reagent Kit (Pierce Biotechnology). The proteins were separated using a 10% sodium dodecyl sulphate polyacrylamide gel electrophoresis (SDS‐PAGE) technique. Subsequently, proteins were translocated onto a polyvinylidene fluoride membrane (Millipore), blocked with 5% bovine serum albumin (BSA) and incubated with primary antibodies, anti‐SPTBN1 (Thermo Fisher Scientific: PA5‐119749), anti‐LC3B (Abcam: ab192890), anti‐NLRP3 (Abcam: ab4207), anti‐ASC (Abcam: ab175449), anti‐cleaved *N*‐terminal GSDMD (Abcam: ab215203 and Affinity Biosciences: DF13758), anti‐Caspase‐1 (Abcam: ab207802) and anti‐IL‐1β (Abcam: ab254360) overnight at 4°C. Subsequently, the membranes were washed and incubated with the appropriate secondary antibodies for 2 h at room temperature. The blots were visualized using the ECL substrate, followed by quantification employing the ImageJ program (NIH Image; Bethesda).

### 2.11 Autophagic flux assessment

The retroviruses encoding tandem‐tagged mCherry‐GFP‐LC3B were prepared to evaluate autophagic flux.^20^ The NCM460 cells were cultivated in a 24‐well plate at 50% confluence and transfected with 1 mL of Ad‐mCherry‐GFP‐LC3 (Beyotime: C3011) overnight. Subsequently, the cell culture medium was substituted with a retrovirus‐containing medium at a MOI of 200 for 60 h. Following a 48‐h treatment with si‐circPRKAR1B, si‐METTL3, or a negative control, the cells were subjected to fixation employing 4% paraformaldehyde, subsequent washing and staining with DAPI. The fluorescence images were acquired utilizing a confocal laser microscope (Leica SP8).

### 2.12 Nuclear cytoplasmic separation

The NE‐PER Nuclear and Cytoplasmic Extraction Reagents (Thermo Scientific) were employed^21^ to isolate the nuclear and cytoplasmic fractions. NCM460 or HCoEpic cells were subjected to an 12‐h stimulation with Alu RNA. Subsequently, the cells were gathered and subjected to lysis employing ice‐cold CER I for 15 min, while maintained on ice. The lysates obtained underwent vortexing and centrifugation at 1000 × *g* for 10 min at 4°C. The pellets were then resuspended in NER, vortexed and centrifuged at 13 000 *g* for 10 min at room temperature. The obtained lysate supernatant was utilized as the nuclear fraction.

### 2.13 RNA extraction and qRT‐PCR

Total RNA extraction was conducted employing the TRIzol reagent (Invitrogen) per the protocols. RNA quality and quantity were assessed utilizing a Nanodrop and a bioanalyzer (Agilent Inc.) Additionally, qRT‐PCR analysis was conducted via the SYBR Green PCR system (Applied Biosystems). The relative circRNA and mRNA expression levels were standardized employing U6 and GAPDH, respectively, and normalized using the 2^−△△^ *^Ct^* technique. The experiments were conducted in triplicate. Table 2 demonstrates the primer sequences employed in this study.

**TABLE 2:** Primers sequences.

Name	Sequence (5′−3′)
Full‐length sequencce of human circPRKAR1B	GAAGGAAGCAGCCACCCTCGCCATGGCCTCCCCGCCCGCCTGCCCCTCGGAGGAGGACGAGAGCCTGAAGGGCTGTGAGCTGTACGTGCAGCTGCACGGGATCCAGCAGGTCCTCAAAGACTGTATCGTCCACCTCTGCATCTCCAAGCCCGAACGCCCCATGAAGTTCCTCCGGGAGCACTTCGAGAAGCTGGAGAAGGAAGAAAACAGGCAGATTTTGGCGCGGCAAAAGTCAAACTCACAGTCGGACTCCCATGATGAGGAGGTGTCGCCCACCCCCCCGAACCCTGTGGTGAAGGCCCGCCGCCGGCGAGGAGGCGTGAGTGCCGAGGTGTACACCGAGGAGGACGCCGTGTCCTACGTCAGGAAGGTGATTCCCAAGGACTACAAAACCATGACTGCGCTGGCCAAGGCCATCTCCAAGAACGTGCTCTTCGCTCACCTGGATGACAACGAGAGGAG
Human circPRKAR1B	
Forward	CAACGAGAGGAGGAAGGAAGC
Reverse	GTCTTTGAGGACCTGCTGGAT
Human SPTBN1	
Forward	TGCATCCAACAAAGAATGGCT
Reverse	GTCTGGGTAGTGGATTCAAGGAC
Human METTL3	
Forward	GATGCTCCTGCCACTCAAGAT
Reverse	GCTTGGAATGGTCAGCATAGG
Human METTL14	
Forward	ACAGAGAAACTGGCATCACT
Reverse	TCGTAAACACACTCTTCCAAGG
Human PRKAR1B	
Forward	CCTGGATGACAACGAGAGGAG
Reverse	CGTTCACGTACACATCCACTTCC
Human WTAP	
Forward	ACTGGCCTAAGAGAGTCTGAAG
Reverse	CATCTCTTGTTCCTTGGTTGCT
Human GAPDH	
Forward	TCCTGGGCTACACTGAGCAC
Reverse	CTGTTGCTGTAGCCAAATTCGTTG
Mouse circPRKAR1B	
Forward	CTTACAAGAGGGGATAATTTAAAG
Reverse	GAGAGGTGTTTGGAAGTCAAG
Mouse METTL3	
Forward	ATATCCGCTACCTGGACGTCA
Reverse	AGCCGTCATCCTGTAGCACTG
Mouse GAPDH	
Forward	GTCAAGGCCGAGAATGGGAA
Reverse	CTCGTGGTTCACACCCATCA
U6	
Forward	ATTGGAACGATACAGAGAAGATT
Reverse	GGAACGCTTCACGAATTTG
Si‐circPRKAR1B‐1 (human)	Target: GTCCTACGTCAGGAAGGTGATTC
Sense	AUCACCUUCCUGACGUAGGAC
Antisense	CCUACGUCAGGAAGGUGAUUC
Si‐circPRKAR1B‐2 (human)	Target:GACAACGAGAGGAGGAAGGAAGC
Sense	UUCCUUCCUCCUCUCGUUGUC
Antisense	CAACGAGAGGAGGAAGGAAGC
Si‐circPRKAR1B‐3 (human)	Target: TCCTACGTCAGGAAGGTGATTCC
Sense	AAUCACCUUCCUGACGUAGGA
Antisense	CUACGUCAGGAAGGUGAUUCC
Si‐METTL3‐1 (human)	Target:CAGACGAATTATCAATAAACACA
Sense	UGUUUAUUGAUAAUUCGUCUG
Antisense	GACGAAUUAUCAAUAAACACA
Si‐METTL3‐2 (human)	Target:GACGAATTATCAATAAACACACT
Sense	UGUGUUUAUUGAUAAUUCGUC
Antisense	CGAAUUAUCAAUAAACACACU
Si‐METTL3‐3 (human)	Target:AAGTATGTTCACTATGAAATTGA
Sense	AAUUUCAUAGUGAACAUACUU
Antisense	GUAUGUUCACUAUGAAAUUGA
Si‐SPTBN1 (human)	Target:TGCTATTGAAACAGAAAAAATGA
Sense	AUUUUUUCUGUUUCAAUAGCA
Antisense	CUAUUGAAACAGAAAAAAUGA
Si‐RNA control	
Sense	UACCCCAGCGGAGGCGUACUA
Antisense	GUACGCCUCCGCTGGGGUAAC
Si‐circPRKAR1B (mouse)	Target: GAGGGGATAATTTAAAGTTGTAG
Sense	ACAACUUUAAAUUAUCCCCUC
Antisense	GGGGAUAAUUUAAAGUUGUAG
Si‐METTL3 (mouse)	Target: GTGCAAGAATTTTGTGATTATGG
Sense	AUAAUCACAAAAUUCUUGCAC
Antisense	GCAAGAAUUUUGUGAUUAUGG
circPRKAR1B FISH probe	5′‐FAM‐GCTGCTTCCTTCCTCCTCTCGTTGTCAT‐3′
circPRKAR1B RNA pull down	
Sense‐F	aaaaTAATACGACTCACTATAGGGGAAGGAAGCAGCCACCCTCG
Sense‐R	CTCCTCTCGTTGTCATCCAGG
Antisense‐F	GAAGGAAGCAGCCACCCTCG
Antisense‐R	aaaaTAATACGACTCACTATAGGGCTCCTCTCGTTGTCATCCAGG

Abbreviations: METTL14, methyltransferase like 14; METTL3, methyltransferase like 3; WTAP, Wilms tumour 1‐associated protein.

### 2.14 RNase R treatment

A total of 10 μg of RNA extracted from NCM460 or HCoEpic cells were incubated with 3 U/μg of RNase R (Epicenter) at 37°C for 30 min. Subsequently, the RNA samples supplemented with RNase R were subjected to reverse transcription utilizing divergent and convergent primers, followed by qRT‐PCR analysis.

### 2.15 RNA stability assay

To assess the RNA decay rate, NCM460 cell lines were exposed to actinomycin D (10 μg/mL). Subsequently, cells were harvested at 0, 12 and 24 h, respectively. Total RNA was purified and subjected to qRT‐PCR analysis. The RNA degradation rate was then calculated relative to the 0‐h time point.

### 2.16 Nucleic acid electrophoresis

The procedure for nucleic acid electrophoresis was carried out following the previously established protocol.^22^ The cDNA and gDNA PCR products were subjected to analysis using the agarose gel electrophoresis technique, employing TAE as the designated running buffer. The electrophoresis process was executed at 100 volts for 30 min to achieve the separation of DNA molecules. Subsequently, the resulting DNA bands were visualized under UV irradiation.

### 2.17 RNA immunoprecipitation (RIP) assay

RIP assays were conducted employing the Magna RIP™ RBP IP kit (Millipore).^23^ Anti‐m^6^A antibody (Sigma: MABE1006) was introduced to the supernatants, and the mixtures were incubated at 4°C overnight. RNA was obtained from Magna RIP beads employing TRIzol Reagent (Invitrogen). Total RNA (input) and isotype control (IgG) for each antibody were assessed simultaneously.

### 2.18 Fluorescence In Situ hybridization (FISH)

The study conducted FISH assays to assess colon tissues and cell lines.^20^ The colon tissues and cell lines were fixed and incubated with 20 μg/mL of proteinase K for 8 min, followed by being pre‐hybridized with pre‐hybridization solution for 60 min at 37°C, hybridized with FAM‐conjugated circRNA probes (300 nM) overnight at 40°C, counterstained with DAPI for 8 min and evaluated employing a Nikon microscope (Eclipse Ti; Nikon).

### 2.19 Oligonucleotide transfection

Prior to oligonucleotide transfection and to stimulate the NLRP3 inflammasome, NCM460 or HCoEpic cells underwent pre‐treatment with LPS at a concentration of 10 ng/mL for 3 h, followed by ATP at a concentration of 5 mM for 30 min (InvivoGen). Afterward, the cells were cultivated into 6‐well plates till they reached 60−70% confluence before transfection with si‐circPRKAR1B, si‐METTL3, or negative control oligonucleotides (Geneseed) employing Lipofectamine™ 2000 reagent (Invitrogen). The primer sequences employed are provided in Table 2.

### 2.20 RNA pull‐down

The cell lysates underwent incubation with streptavidin‐coated magnetic beads to isolate the biotin‐coupled RNA complex, following the protocols. The captured fractions were assessed for circPRKAR1B or SPTBN1 enrichment through qRT‐PCR analysis. The bead‐attached proteins were eluted and subsequently analyzed using SDS‐PAGE. The study employed silver staining, mass spectrometry (MS) analysis and western blotting to identify the captured complex proteins. Table 2 lists the circPRKAR1B junction probes (Ruqi Biotech).

### 2.21 Silver staining and MS analysis

The silver staining procedure was conducted using the Fast Silver Stain Kit (Beyotime) per the instructions. The proteins were identified and quantified using PEAKS software (Bioinformatics Solutions, Inc.). A protein with an area ratio (sense/anti‐sense FC) > 2 and unique peptides >2 was deemed significant.

### 2.22 Statistical analyses

Statistical analyses were conducted utilizing SPSS 19.0 software (Chicago) and GraphPad Prism 8.0 (San Diego). Data analysis was conducted using the Student's *t*‐test or one‐way analysis of variance (ANOVA). Associations between relative circPRKAR1B expression and inflammatory indices (CRP, CDAI and SES‐CD) were evaluated by Pearson's correlation analysis. The statistical significance level was established at a significance level of *p* < .05.

## 3 RESULTS

### 3.1 circRNA screening and circPRKAR1B verification

**FIGURE 1:**
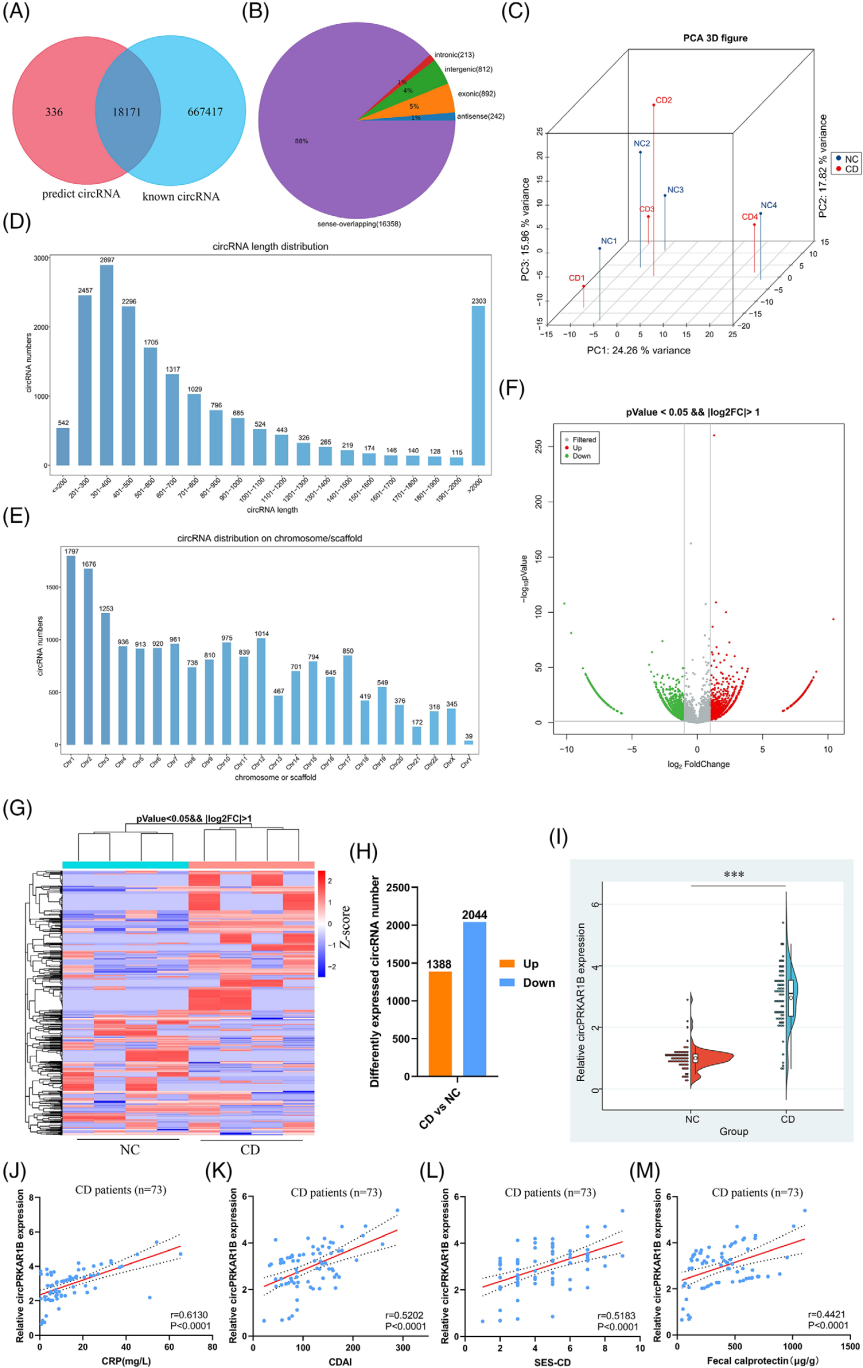
The circPRKAR1B as circRNA candidate. (A) Overlap of circRNAs in RNA‐seq (left) and circBase (right). (B) Composition of circRNAs in terms of genomic origin. (C) Correlations between samples according to the 3D PCA plot distribution. (D) Length distribution of detected circRNAs. (E) CircRNA distribution on chromosomes/scaffolds. Volcano plots (F), heatmap (G) and histogram (H) showing circRNA expression profiles in colon tissues from CD patients and NCs according to RNA‐seq (threshold: *p* < .05 and |log2FC| > 1). (I) Relative circPRKAR1B expression in CD patients and NCs according to qRT‐PCR analysis. Correlations between circPRKAR1B levels and CD‐associated inflammatory indicators, including CRP (J), CDAI (K), SES‐CD (L) and faecal calprotectin (M), in CD patients. circRNA, circular RNA; PCA, principal component analysis; CD, Crohn's disease; NC, normal control; FC, fold change; qRT‐PCR, quantitative real‐time polymerase chain reaction; CRP, C‐reactive protein; CDAI, Crohn's disease activity index; SES‐CD, simple endoscopic score for Crohn's disease. The data are presented as mean ± SD. ***p < .001 by Student's *t*‐tests.

The circRNA‐seq analysis was performed on eight colon samples (four CD vs. four NC) to investigate the role of circRNAs in CD, detecting 18 507 distinct circRNAs in all samples, reporting 18 171 out of them (Figure 1A). Figure 1B shows the composition of the expressed circRNAs in terms of genomic origin. The principal component analysis (PCA) 3D distribution plot indicated a close distribution between CD and control sample sets (Figure 1C). The circRNA length distribution was quite different, and most were less than 1000 nucleotides in length (Figure 1D). Figure 1E displays the circRNA distribution on the chromosome or scaffold. The volcano plot (Figure 1F) and cluster heatmap (Figure 1G) present the significantly dysregulated circRNAs in CD tissues compared to NC tissues with thresholds of *p* < .05 and a |log_2_FC| > 1. A total of 1388 out of these 3432 analysed circRNAs, were upregulated, while 2044 were downregulated in the CD tissues (Figure 1H). After further screening (|log_2_FC| > 2 and expression detected in all samples), we focused on the most upregulated hsa_circ_0008039 (circPRKAR1B). To further verify circPRKAR1B expression, we expanded the experimental sample size (73 CD vs. 73 NC). The qRT‐PCR revealed a significantly elevated expression in CD individuals compared to NCs (Figure 1I). Additionally, relative circPRKAR1B was closely correlated to CD inflammatory indices, including CRP (Figure 1J), CDAI (Figure 1K), SES‐CD (Figure 1L) and faecal calprotectin (Figure 1M), in 73 CD patients. All these results strongly indicated that circPRKAR1B is a circRNA closely associated with CD.

### 3.2 Characterization of circPRKAR1B

**FIGURE 2:**
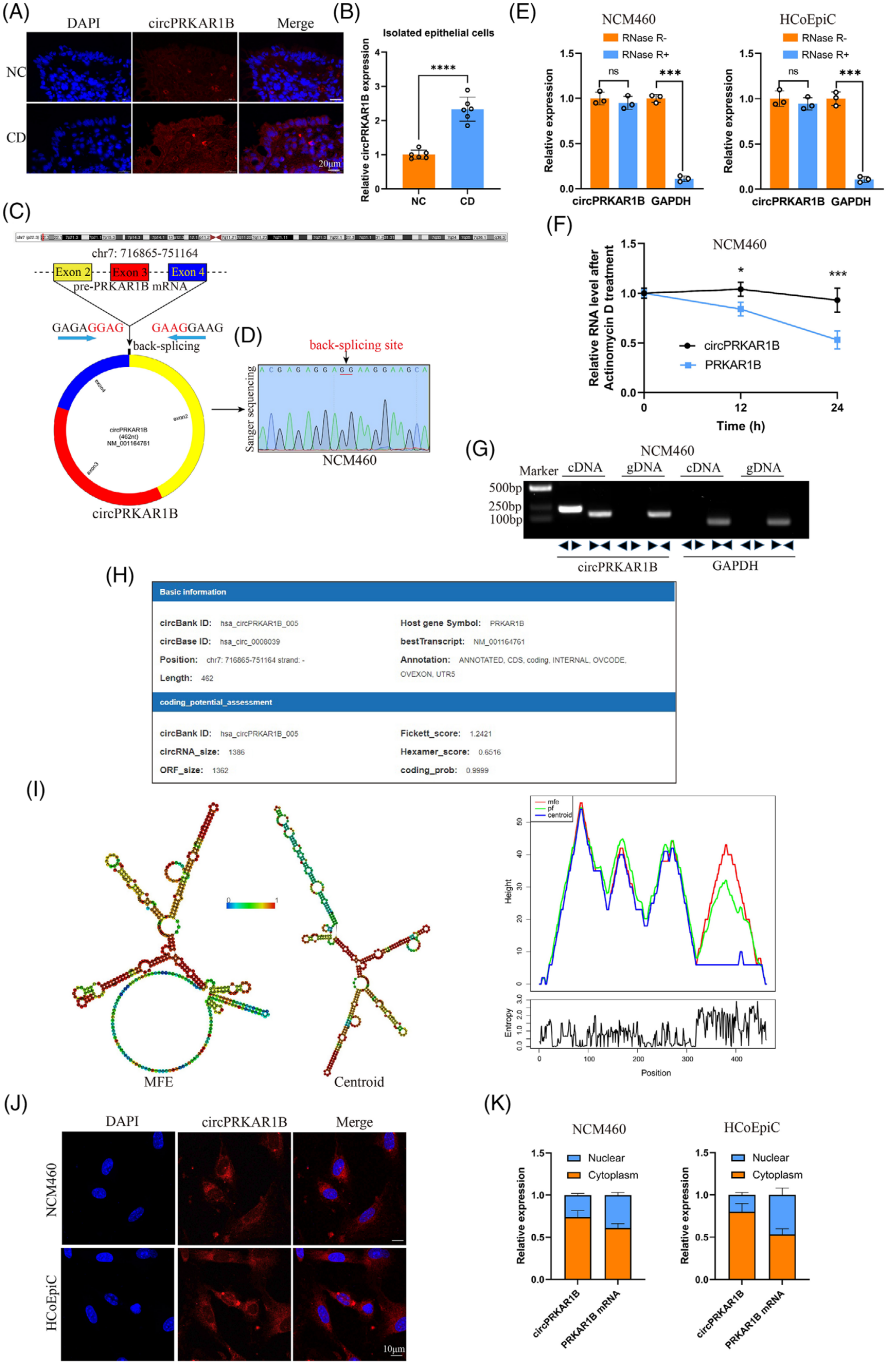
Characterization of circPRKAR1B. (A) FISH analysis of circPRKAR1B in colon tissues from CD patients and NCs; scale bar: 20 μm. (B) Relative circPRKAR1B expression in isolated epithelial cells from CD patients and NCs according to qRT‐PCR. (C) The circular structure of circPRKAR1B back‐spliced by exons 2 and 4. (D) Sanger sequencing confirmed the circular structure in NCM460 cells. (E) RNase R treatment experiments with qRT‐PCR in NCM460 and HCoEpic cell lines. Actinomycin D assay (F) and agarose gel electrophoresis (G) analyses in NCM460 cell lines. (H) Basic information on circPRKAR1B from the circBank database. (I) The predicted secondary structures of circPRKAR1B based on the RNAfold website. (J) Localization of circPRKAR1B in NCM460 and HCoEpic cell lines according to FISH analysis; scale bar: 10 μm. (K) Expression of circPRKAR1B in the cytoplasm and nucleus according to qRT‐PCR. circRNA, circular RNA; CD, Crohn's disease; NC, normal control; qRT‐PCR, quantitative real‐time polymerase chain reaction; MFE, minimum free energy; FISH, fluorescence In Situ hybridization; ns, non‐significant, ***p < .001, ****p < .0001 by Student's *t*‐tests.

The FISH results indicated that circPRKAR1B was upregulated in CD colon tissues compared to paired NC tissues and mainly localized in the epithelial layers (Figure 2A). Further quantitative analysis showed that circPRKAR1B expression was significantly higher in isolated epithelial cells of CD individuals than in NCs (Figure 2B). The data obtained from the circBank and University of California, Santa Cruz (UCSC) genome browser databases revealed that circPRKAR1B originates from the PRKAR1B gene and situated at the chr7 (716865‐751164). The structure is formed through back‐splicing involving exons 2 and 4, with a 462 nucleotide length (Figure 2C). The looped circPRKAR1B structure was constructed employing circPrimer 2.0 software^24^ (Figure 2C), and the sequence containing the back‐splicing site was validated by Sanger sequencing using the NCM460 cell line (Figure 2D). Furthermore, RNase R experiments indicated that circPRKAR1B is resistant to RNase R treatment in NCM460 and HCoEpic cell lines (Figure 2E). The actinomycin D assay confirmed the good stability of circPRKAR1B in NCM460 cell lines (Figure 2F). In the cDNA and gDNA analyses in NCM460 cell lines, circPRKAR1B exhibited amplification exclusively by divergent primers in cDNA (Figure 2G). Figure 2H illustrates the basic information and coding potential of circPRKAR1B based on the circBank database. The RNA‐fold algorithm was utilized to determine the predicted minimum free energy (MFE) and centroid secondary structures of circPRKAR1B^25^ (Figure 2I). Subcellular localization analysis by FISH showed that circPRKAR1B was primarily in the cytoplasm of NCM460 and HCoEpic cell lines (Figure 2J). The qRT‐PCR analysis outcomes indicated that relative circPRKAR1B expression in the cytoplasm was significantly elevated compared to the nucleus (Figure 2K).

### 3.3 circPRKAR1B is regulated by METTL3‐mediated m^6^A methylation

**FIGURE 3:**
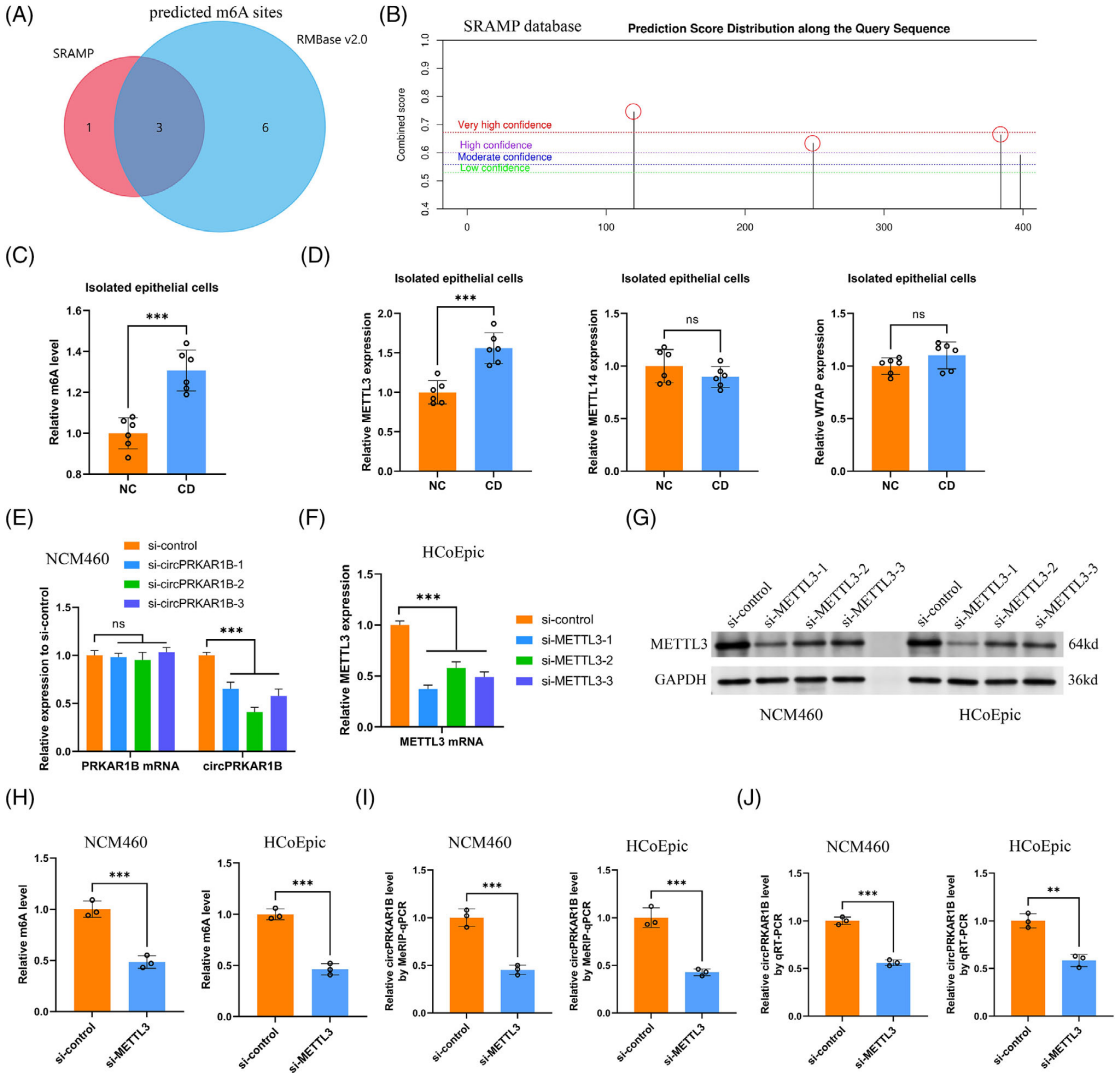
circPRKAR1B is regulated by METTL3‐mediated m^6^A methylation. (A) m^6^A methylation modification sites predicted by SRAMP and RMBase 2.0. (B) m^6^A sites predicted with high confidence by SRAMP. (C) Relative m^6^A levels in isolated epithelial cells from CD patients and NCs. (D) Expression of m^6^A methyltransferases, including METTL3/14 and WTAP, in isolated epithelial cells from CD patients and NCs. (E) Impacts of three si‐circPRKAR1B sequences on the expression of circPRKAR1B and PRKAR1B mRNA according to qRT‐PCR. Efficacy of three designed si‐METTL3 sequences according to qRT‐PCR (F) and western blotting (G). The impacts of si‐METTL3 on m^6^A levels according to qRT‐PCR (H) and circPRKAR1B expression according to MeRIP‐qPCR (I) and qRT‐PCR (J) were determined in NCM460 and HCoEpic cell lines. METTL3, methyltransferase‐like 3; METTL14, methyltransferase‐like 14; m^6^A, N6‐methyladenosine; CD, Crohn's disease; NC, normal control; WTAP, Wilms tumour 1‐associated protein; qRT‐PCR, quantitative real‐time polymerase chain reaction; RIP, RNA immunoprecipitation. The data are presented as mean ± SD. ***p < .001 by Student's *t*‐tests.

To investigate the potential explanations for the abnormal circPRKAR1B overexpression, we focused on the m^6^A modification of circPRKAR1B. Based on the circBank database, m^6^A modification existed on circPRKAR1B. The predicted m^6^A sites on its sequences were listed using the SRAMP^26^ and RMBase V2.0^27^ online databases (Figure 3A and B). The m^6^A levels were significantly elevated in epithelial cells of CD individuals compared to NCs (Figure 3C). The methyltransferases like methyltransferase‐like 3 (METTL3), METTL14 and Wilms' tumour‐1‐associated protein (WTAP), are key catalysts of the methylation process (m^6^A writers).^28^ Our outcomes showed that only METTL3 levels were significantly higher in the isolated epithelial cells from CD individuals than NCs (Figure 3D). Therefore, three siRNA sequences for circPRKAR1B and METTL3 were designed and synthesized, revealing that si‐circPRKAR1B‐2 (Figure 3E) and si‐METTL3‐1 (Figure 3F and G) demonstrated the most significant inhibitory effectiveness. Consequently, these siRNAs were used for further experiments. Additionally, the relative m^6^A level was significantly decreased by si‐METTL3 treatment in NCM460 and HCoEpic cell lines (Figure 3H). Relative circPRKAR1B expression determined by MeRIP followed by qRT‐PCR was significantly decreased by si‐METTL3 treatment (Figure 3I). Moreover, circPRKAR1B levels were suppressed by si‐METTL3 treatment (Figure 3J). Collectively, our outcomes strongly indicated that METTL3 at least partially mediated the m^6^A modification of circPRKAR1B.

### 3.4 circPRKAR1B interacts with SPTBN1

**FIGURE 4:**
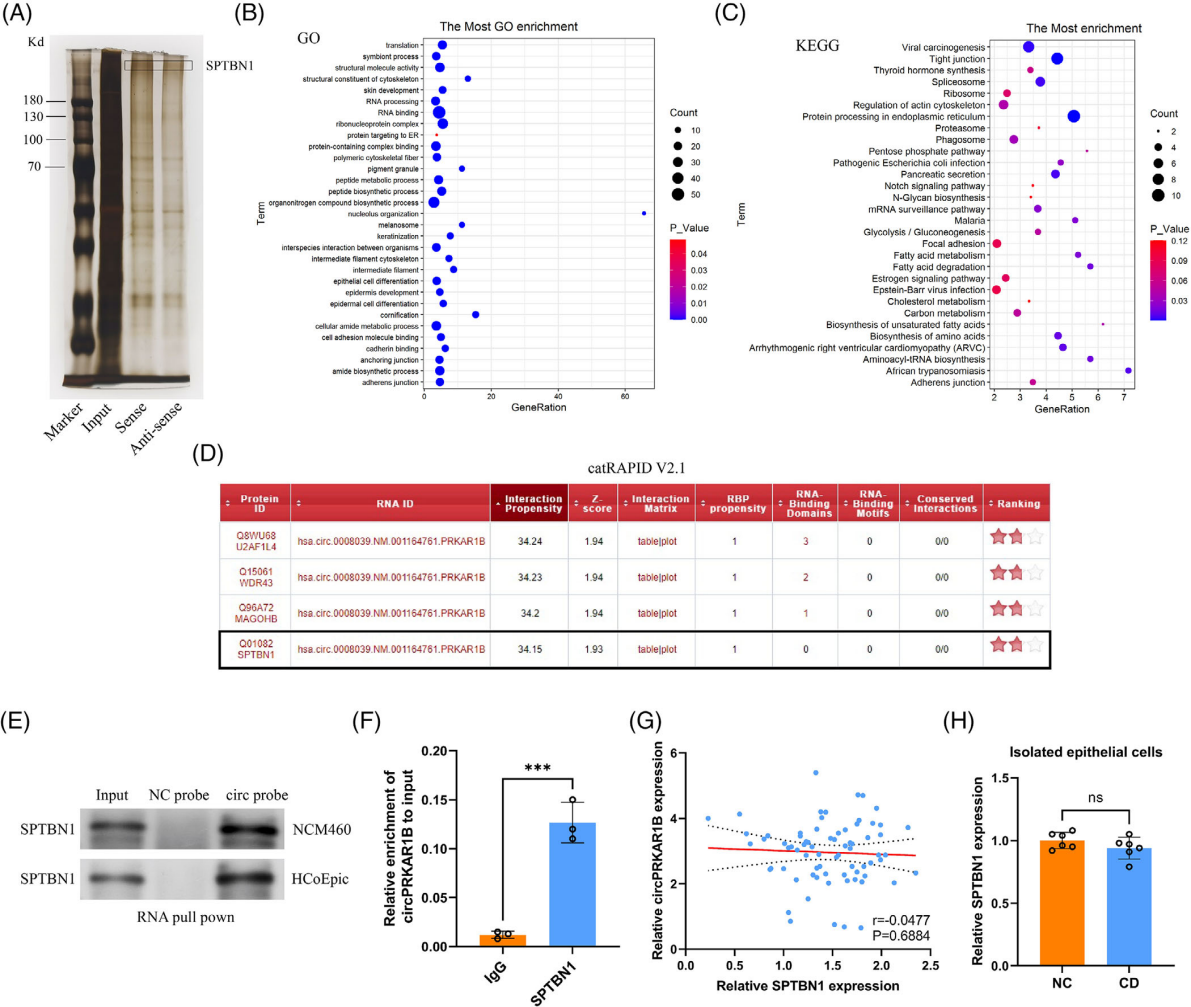
circPRKAR1B interacts with SPTBN1. (A) Silver staining of the RNA pull‐down assay results. According to MS analysis of the RNA pull‐down products, deregulated proteins were subjected to GO (B) and KEGG enrichment (C) analyses. (D) Binding potential between circPRKAR1B and SPTBN1 by the catRAPID database. The interaction assessment between circPRKAR1B and SPTBN1 by RNA pull‐down western blotting (E) and RIP assays (F). (G) Correlation between circPRKAR1B and SPTBN1 levels in colon tissues from CD patients. (H) Relative SPTBN1 expression in isolated epithelial cells from CD patients and NCs. SPTBN1, spectrin beta, non‐erythrocytic 1; GO, Gene Ontology; KEGG, Kyoto Encyclopedia of Genes and Genomes; CD, Crohn's disease; NC, normal control; RIP, RNA immunoprecipitation. The data are expressed as mean ± SD; ns, non‐significant, ***p < .001 by Student's *t*‐tests.

The circRNAs exert their functions by binding to RBP.^29^, ^30^ To identify the interacting RBPs, we performed an RNA pull‐down assay with NCM460 cell lysates using biotinylated circPRKAAR1B sense, antisense and negative control probes. Subsequent silver staining (Figure 4A) and MS analysis identified SPTBN1 (ranked in the top five, see the supporting information) as a potential circPRKAR1B‐binding protein. The deregulated proteins by MS analysis were then subjected to enrichment analyses of Gene Ontology (Figure 4B) and Kyoto Encyclopedia of Genes and Genomes (KEGG) (Figure 4C). Additionally, the catRAPID online database supported the binding potential between circPRKAR1B and SPTBN1 (Figure 4D). It was validated in NCM460 and HCoEpic cell lines that abundant SPTBN1 could be pulled down by circPRKAR1B (Figure 4E). Furthermore, RIP assays indicated that circPRKAR1B could be enriched by SPTBN1 (Figure 4F). Collectively, the findings indicated that SPTBN1 could bind to circPRKAR1B. Additionally, no linear connection was detected between relative circPRKAR1B and SPTBN1 expression in the colon tissues of CD individuals (Figure 4G). SPTBN1 expression in isolated epithelial cells did not differ between CD patients and NCs (Figure 4H).

### 3.5 In vitro function of circPRKAR1B

**FIGURE 5:**
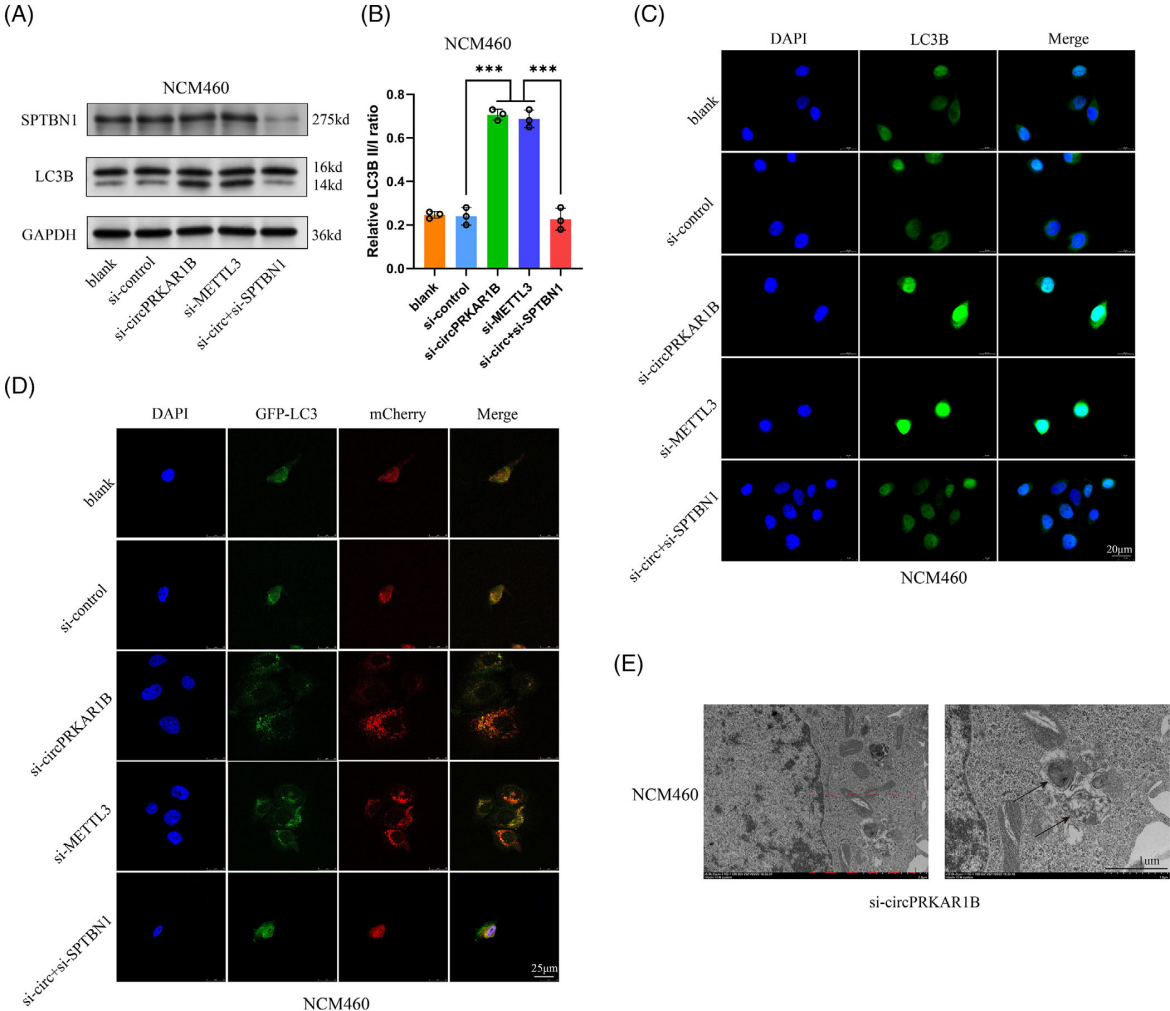
Impacts of circPRKAR1B and m^6^A modification on cell autophagy. The assessment of the impacts of si‐circPRKAR1B, si‐METTL3 and si‐circ + si‐SPTBN1 treatment on epithelial cell autophagy by western blotting (A), grayscale analysis of LC3B II/I (B), immunofluorescence (C; scale bar: 20 μm), autophagic flux analysis via mCherry‐GFP‐LC3B confocal microscopy (D; scale bar: 25 μm) and autophagosome analysis via TEM (E; scale bar: 1 μm). METTL3, methyltransferase‐like 3; m^6^A, N6‐methyladenosine; TEM, transmission electron microscope. The data are presented as mean ± SD. ***p < .001 by one‐way ANOVA.

**FIGURE 6:**
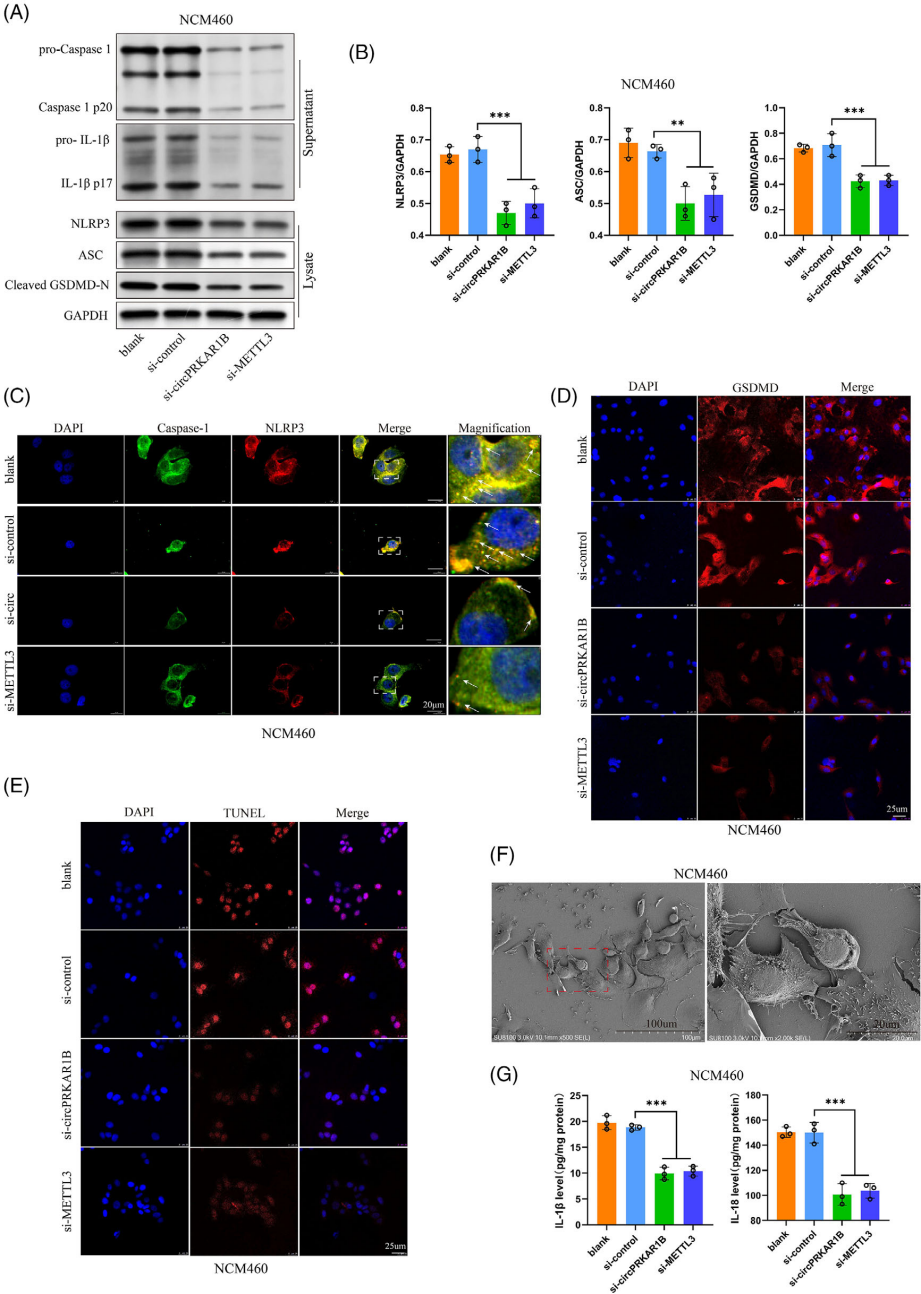
Impacts of circPRKAR1B and m^6^A modification on NLRP3 inflammasome‐mediated pyroptosis. The assessment of the impacts of si‐circPRKAR1B and si‐METTL3 treatment on NLRP3 inflammasome‐mediated pyroptosis using western blotting (A), grayscale analysis of blots (B), immunofluorescence (C; scale bar: 20 μm, white arrows: NLRP3 inflammasome specks and D; scale bar: 25 μm), TUNEL staining (E; scale bar: 25 μm), pyroptosis analysis via SEM (F; scale bar: 100 and 20 μm) and proinflammatory cytokine (IL‐1β/18) level analyses via ELISA (G). METTL3, methyltransferase‐like 3; m^6^A, N6‐methyladenosine; SEM, scanning electron microscopy; NLRP3, nucleotide‐binding oligomerization domain, leucine‐rich repeat and pyrin domain‐containing 3; TUNEL, terminal deoxynucleotidyl transferase dUTP nick end labeling; ELISA, enzyme‐linked immunosorbent assay; IL, interleukin. The data are presented as mean ± SD. ***p < .001 by one‐way ANOVA.

SPTBN1 loss suppresses autophagy by modulating Yes‐associated protein (YAP) methylation in liver cell carcinoma,^31^ indicating a positive association between SPTBN1 and autophagy. Autophagy has been shown to be related to CD pathogenesis.^32^, ^33^ Additionally, autophagy can modulate pyroptosis mediated by the NLRP3 inflammasome,^34^ which is strictly connected with the IL‐1β‐mediated inflammatory response.^35^, ^36^ NCM460 cells were incubated with LPS and ATP to activate the NLRP3 inflammasome. The autophagy level was effectively upregulated by si‐circPRKAR1B and si‐METTL3 treatment, as exhibited by an increased ratio of LC3B II/I in western blotting (Figure 5A and B), immunofluorescence (Figure 5C), autophagic flux determined by mCherry‐GFP‐LC3B confocal microscopy (Figure 5D) and autophagosome analysis according to TEM (Figure 5E). All these results verified the promoting roles of si‐circPRKAR1B and si‐METTL3 in cell autophagy. In addition, si‐SPTBN1 reversed the phenotypes caused by si‐circPRKAR1B treatment (Figure 5A‐D), indicating the association between SPTBN1 with autophagy. However, SPTBN1 expression was not influenced by si‐circPRKAR1B or si‐METTL3 treatment (Figure 5A). Additionally, si‐circPRKAR1B and si‐METTL3 treatment significantly inhibited LRP3 inflammasome‐mediated pyroptosis, as shown by decreased active Caspase‐1, IL‐1β, NLRP3, ASC and cleaved N‐terminal GSDMD expressions in western blot analysis (Figure 6A and B) and immunofluorescence analysis (Figure 6C and D), decreased apoptosis according to TUNEL staining (Figure 6E), pyroptosis according to SEM (Figure 6F) and decreased proinflammatory cytokine levels (IL‐1β/18) (Figure 6G).

**FIGURE 7:**
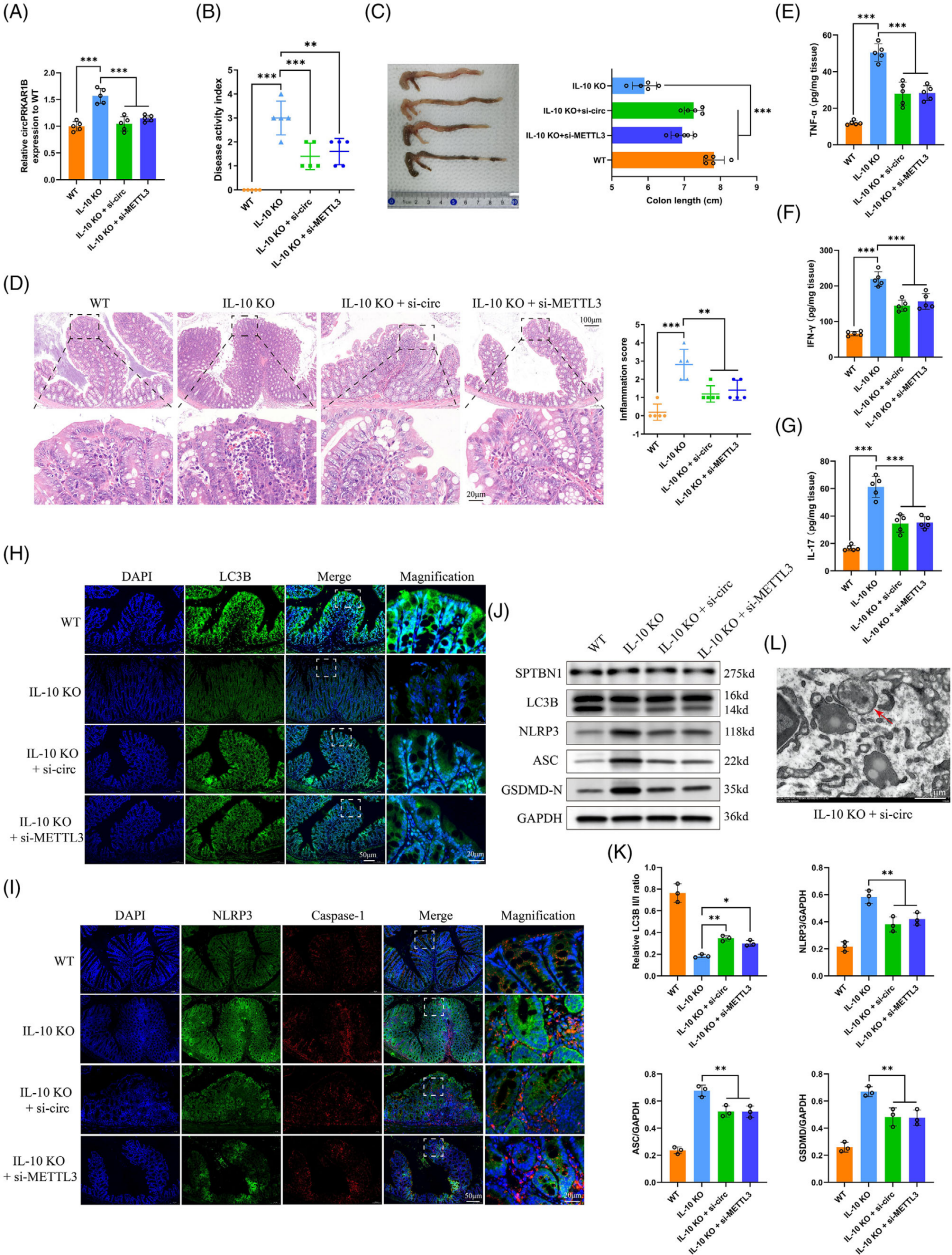
In vivo circPRKAR1B and m^6^A modification functions in IL‐10‐KO mice. The assessment of the impacts of si‐circPRKAR1B and si‐METTL3 treatment on the relative circPRKAR1B expression (A), disease activity index (B), colon length (C), pathological inflammatory score from HE staining (D) and inflammatory cytokines including TNF‐α (E), IFN‐γ (F) and IL‐17 (G). The impacts of si‐circPRKAR1B and si‐METTL3 treatment on autophagy and NLRP3 inflammasome‐mediated pyroptosis were assessed by immunofluorescence (H and I; scale bar: 50 and 20 μm), western blotting (J), grayscale analysis of blots (K) and autophagosome analysis via TEM (L; scale bar: 1 μm). SPTBN1, spectrin beta, non‐erythrocytic 1; KO, knockout; METTL3, methyltransferase‐like 3; m^6^A, N6‐methyladenosine; HE, haematoxylin and eosin; TEM, transmission electron microscopy; TNF‐α, tumour necrosis factor‐α; IFN‐γ, interferon‐γ; IL‐17, interleukin‐17; NLRP3, nucleotide‐binding oligomerization domain, leucine‐rich repeat and pyrin domain‐containing 3. The data are presented as mean ± SD. *p < .05, **p < .01, ***p < .001 by one‐way ANOVA.

We further explored the in vivo functions of circPRKAR1B using IL‐10‐KO mice as the colitis model. Relative circPRKAR1B expression was significantly elevated in IL‐10‐KO mice compared to WT mice. The si‐circPRKAR1B and si‐METTL3 treatments significantly suppressed the circPRKAR1B level (Figure 7A). Colonic inflammation was significantly improved. This was evident by decreased DAI values (Figure 7B), improved colon length shortening (Figure 7C), decreased histological inflammation scores (Figure 7D) and decreased inflammatory cytokines levels including TNF‐α (Figure 7E), IFN‐γ (Figure 7F) and IL‐17 (Figure 7G). Furthermore, we investigated the impacts of circPRKAR1B knockdown on autophagy and pyroptosis mediated by the NLRP3 inflammasome in experimental colitis models. The level of autophagy was significantly improved by si‐circPRKAR1B and si‐METTL3 treatment. This was evident by improved LC3B expression and distribution in immunofluorescence analysis (Figure 7H), an increased ratio of LC3B II/I in western blot analysis (Figure 7J and K), and increased autophagosome numbers in TEM analysis (Figure 7L). Additionally, pyroptosis mediated by the NLRP3 inflammasome was inhibited by si‐circPRKAR1B and si‐METTL3 treatment, as shown by the downregulated‐associated proteins (Figure 7I–K). These outcomes indicated that the anti‐inflammatory functions of si‐circPRKAR1B and si‐METTL3 were mediated by inhibiting pyroptosis via autophagy induction.

**FIGURE 8:**
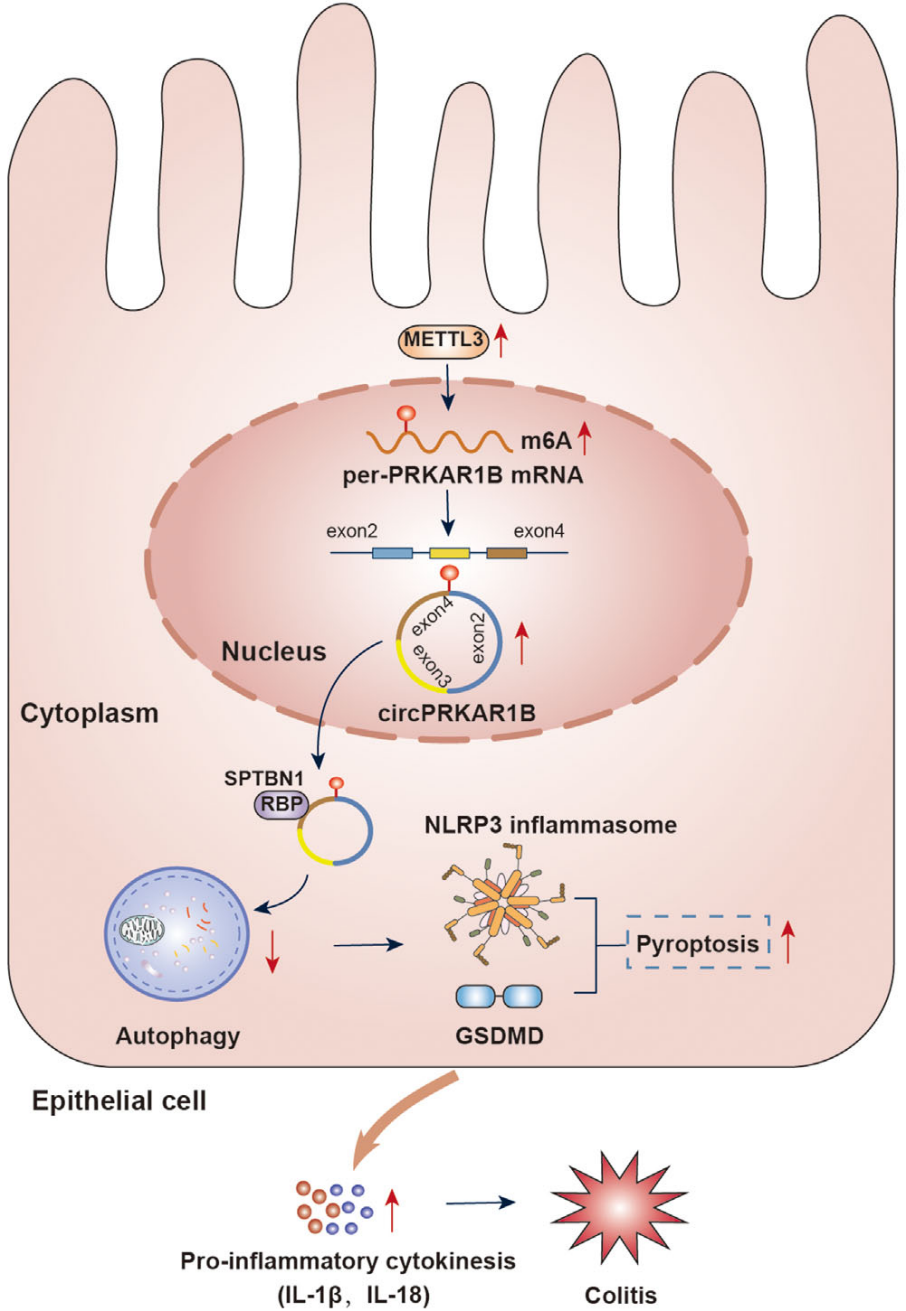
Graphical illustration of how METTL3‐mediated m^6^A modification of circPRKAR1B promotes Crohn's colitis by inducing pyroptosis via autophagy inhibition. SPTBN1, spectrin beta, non‐erythrocytic 1; METTL3, methyltransferase‐like 3; m^6^A, N6‐methyladenosine; HE, haematoxylin and eosin; IL‐1β, interleukin‐1β; NLRP3, nucleotide‐binding oligomerization domain, leucine‐rich repeat and pyrin domain‐containing 3; RBP, RNA‐binding protein.

Our study indicated that aberrantly circPRKAR1B overexpression induced by METTL3‐mediated m^6^A modification impaired autophagy and pyroptosis activation by interacting with the RBP SPTBN1, aggravating Crohn's colitis (Figure 8).The RNA‐seq and bioinformatic analysis progress have detected multiple circRNAs in many diseases and have received extensive attention.^37^ The functions of most circRNAs in physiology and disease progression remain unexplored despite their high stability, evolutionary conservation and tissue‐specific characteristics.^38^ Herein, we screened hundreds of differentially expressed circRNAs in colon tissues and found that circPRKAR1B acts as a promoter of colonic inflammation by enhancing NLRP3 inflammasome‐mediated pyroptosis and suppressing cell autophagy via interaction with SPTBN1. PRKAR1B, the origin gene of circPRKAR1B, exhibited no significant differences in colon samples or isolated epithelial cells between the CD and NC groups. Furthermore, no associations were observed between PRKAR1B and colitis or inflammation. Therefore, circPRKAR1B affects CD progression independently of PRKAR1B. Additionally, highly expressed circPRKAR1B was at least partly dependent on METTL3‐mediated m^6^A modification. These findings link circRNA with the pathogenesis of Crohn's colitis and provide a probable novel biological treatment target.

The circPRKAR1B can promote hepatic malignancy development through the miR‐432‐5p/E2F3 mechanism.^39^ This study first identified the biological roles and molecular mechanisms of circPRKAR1B in Crohn's colitis. CircRNAs regulate gene expression through various mechanisms,^8^ particularly direct or indirect interaction with RBPs.^30^ SPTBN1 was verified as an RBP interacting with circPRKAR1B based on RNA pull‐down, MS analysis and RIP assays. Chen et al.^31^ showed that SPTBN1 cooperated with the inhibitor of variegation 3‐9‐enhancer of zeste‐trithorax domain comprising lysine methyltransferase 7 (SETD7) to facilitate the methylation of YAP. This cooperation deactivated YAP and induced autophagy in hepatocellular carcinoma cells.

Moreover, loss of SPTBN1 in HCC cells increases the stability of the p65 protein by inhibiting SOCS1 and enhancing NF‐κB activation, releasing the inflammatory cytokines, IL‐1α/1β/6.^40^ Our outcomes indicated that SPTBN1 expression level was unaffected by circPRKAR1B overexpression or knockdown, suggesting that SPTBN1 binding decreases the levels of free SPTBN1. This reduction impaired autophagy and increased colonic inflammation. Our in vivo and in vitro outcomes confirm that circPRKAR1B influences autophagy by interacting with SPTBN1. However, the underlying molecular mechanisms remain elusive and require further investigation. Besides interacting with RBPs, circRNAs can function through different pathways by serving as miRNA sponges and regulating transcription and translation. This study explored the underlying mechanisms of circPRKAR1B by examining the interactions between circRNA with RPBs. However, whether circPRKAR1B functions through other mechanisms, including the ceRNA mechanism, remain unclear. Additionally, this study focused on SPTBN1, which is among the most promising RBPs that interact with circPRKAR1B. We hypothesize that circPRKAR1B may interact with other RBPs and contribute to Crohn's colitis via alternative pathways.

Autophagy, a membrane‐depolarizing clearance process that occurs after external stimuli or aging, is a normal self‐repair mechanism of cells.^41^ The imbalance of autophagy is connected with various disorders, and the imbalance in intestinal epithelial cells triggers intestinal immune disorders and inflammatory processes.^42^ Autophagy is involved in CD by participating in the T‐cell immune response and tolerance, besides regulating intracellular bacteria clearance, NF‐κB activation and inflammasome activity.^43^ Our outcomes revealed that inhibiting pyroptosis caused by NLRP3 inflammasome was an important mechanism by which circPRKAR1B knockdown alleviated Crohn's colitis via targeting autophagy.

m^6^A alteration is the most prevalent epigenetic modification among the several post‐transcriptional RNA modifications,^44^ significantly affecting precursor shearing, nuclear export, RNA translation and RNA stability.^45^ m^6^A modification is prevalent in circRNAs.^46^ METTL3, a well‐known m^6^A modification writer, is a necessary catalytic subunit modulating m^6^A modification.^47^ METTL3 was upregulated in CD colon tissues compared to NC tissues, consistent with Yang et al.^48^ The close m^6^A modification and inflammation connection have attracted much attention. A previous study in human dental pulp cells has shown that METTL3 can suppress the inflammatory response caused by LPS by modulating the alternate splicing of MyD88.^49^ Moreover, METTL3 enhanced microglial inflammation caused by LPS by modulating the TRAF6/NF‐κB mechanism.^50^ Additionally, METTL3 deletion in mouse T cells disrupted T‐cell homeostasis and differentiation.^51^ Furthermore, T cells lacking METTL3 could not undergo homeostatic expansion in a mouse model of adoptive transfer‐induced lymphopaenia, ultimately preventing colitis.^51^ All these results support the proinflammatory functions of METTL3‐mediated m^6^A modification, consistent with our results. The process of m^6^A modification is regulated by several factors, including readers (YTHDF1/2/3 and YTHDC1/2, among others), writers (METTL3/14 and WTAP) and erasers (FTO and ALKBH5), as well as auxiliary regulatory factors including IGF2BP.^52^ This work focused on the crosstalk between methylation writers and m^6^A modification. Our findings indicate that METTL3, rather than METTL14 and WTAP, can modify circPRKAR1B and facilitate its nucleation process. However, it remains unclear whether other methylation‐related proteins (including ALKBH5 and YTHDC1) regulate circPRKAR1B, which warrants further investigation. Additionally, we validated that m^6^A modification has a significant function in the aberrant upregulation of circPRKAR1B. Nevertheless, other factors, including transcription factor binding and alternative splicing regulation, may also contribute to the elevated expression of circPRKAR1B, which requires further investigation.

## 4 CONCLUSIONS

Our study reveals that circPRKAR1B upregulation, which is at least partially induced by METTL3‐mediated m^6^A modification, promotes Crohn's colitis by aggravating NLRP3 inflammasome‐mediated pyroptosis via autophagy impairment.

## CONFLICT OF INTEREST STATEMENT

The authors declare no conflict of interest.

## FUNDING INFORMATION

This work was supported by funding from the ).

## Supporting information

Supporting Information

## Data Availability

Please contact the corresponding author (Liming Tang) upon reasonable request.
